# Drug-Induced Myocardial Infarction: A Review of Pharmacological Triggers and Pathophysiological Mechanisms

**DOI:** 10.3390/jcdd11120406

**Published:** 2024-12-18

**Authors:** Ioana Boarescu, Paul-Mihai Boarescu

**Affiliations:** 1 Neurology Department, Clinical Emergency County Hospital Saint John the New, 720229 Suceava, Romania; 2 Cardiology Departement, Clinical Emergency County Hospital Saint John the New, 720229 Suceava, Romania; 3 Department of Medical-Surgical and Complementary Sciences, Faculty of Medicine and Biological Sciences, “Stefan cel Mare” University of Suceava, 720229 Suceava, Romania

**Keywords:** drugs, myocardial infarction, pharmacological triggers, pathophysiological mechanisms

## Abstract

Myocardial infarction (MI) is a significant cardiovascular event caused by the decrease in or complete cessation of blood flow to a portion of the myocardium. It can arise from a variety of etiological factors, including pharmacological triggers. This review aims to explore the diverse drugs and substances that might lead to drug-induced myocardial infarction, focusing on their mechanisms of action and the pathophysiological processes involved. Various established and emerging pharmacological agents that could elevate the risk of myocardial infarction, such as nonsteroidal anti-inflammatory drugs, hormonal therapies, anticoagulants, and antipsychotic medications, are discussed. The role of drug-induced endothelial dysfunction, coronary artery spasm, and thrombosis are presented in order to highlight the underlying mechanisms. This review emphasizes the need for increased awareness among healthcare professionals to mitigate the risks associated with different pharmacological therapies to improve patient outcomes.

## 1. Myocardial Infarction

Myocardial infarction (MI) is pathologically defined as the death of myocardial cells as a result of prolonged ischemia. In the case of an MI, the necrosis extends from the subendocardium to the subepicardium over several hours. This progression can be delayed by factors such as decreased determinants of myocardial oxygen consumption, intermittent occlusion/reperfusion, and increased collateral blood flow, which can condition the heart. Reperfusion therapy, when indicated, initiated as soon as possible, can further reduce ischemic damage to the myocardium [1,2].

### 1.1. Biomarker Detection of Myocardial Injury and Infarction

Cardiac troponin I (cTnI) and troponin T (cTnT) are critical components of the contractile apparatus of myocardial cells and are predominantly expressed in the cardiac tissue. cTnI and cTnT are considered primary biomarkers for assessing myocardial injury, with high-sensitivity (hs)-cTn tests recommended for routine clinical application. Increases in cTnI levels are often associated with cardiac tissue damage. For cTnT, the situation is more complex, as biochemical evidence suggests that injured skeletal muscle may produce detectable proteins via the cTnT test. Other biomarkers, such as the MB isoform of creatine kinase (CK-MB), are considered less sensitive and specific [3,4].

Myocardial injury is defined by increased blood cTn levels exceeding the 99th percentile upper reference limit (URL) [3,4,5]. While elevated cTn levels indicate damage to myocardial cells, they do not reveal the underlying pathophysiological mechanisms. Such elevations may result from preload-induced mechanical strain or physiological stress in otherwise healthy hearts. However, it remains challenging to identify the specific mechanisms responsible for elevated cTn levels [3,5]. Other organs may also be affected, especially the liver. Advanced imaging techniques, such as cardiovascular magnetic resonance, can help diagnose hepatic alterations secondary to acute myocardial infarction [6].

### 1.2. Clinical Presentations of Myocardial Infarction

The onset of myocardial ischemia is the first step toward MI due to an imbalance between oxygen demand and supply [7]. In clinical practice, myocardial ischemia is often identified through the ECG changes correlated to the patient’s clinical presentation and medical history.

The symptoms of myocardial ischemia can be presented as various combinations of chest pain radiating to the upper extremities, jaw, or epigastric area, occurring during physical exertion or even at rest. Patients may also experience fatigue or dyspnea. However, these symptoms are not exclusive to myocardial ischemia and may occur in other gastrointestinal, pulmonary, musculoskeletal, or neurological conditions [8].

Patients with MI may also be asymptomatic or present with atypical symptoms, such as palpitations, sweating, or even cardiac arrest [2]. Short episodes of ischemia, which are too short to cause necrosis, can still lead to the release of cTn into the bloodstream and an increase in cTn serum levels, with the affected myocytes potentially undergoing apoptosis [9].

### 1.3. Clinical Classification of Myocardial Infarction

Identifying myocardial infarction in patients presenting with ST-segment elevation (STEMI) on ECG or new bundle branch blocks is essential to facilitating immediate treatment strategies, such as reperfusion therapy. Those without ST-segment elevation are classified as non-ST-elevation MI (NSTEMI).

Further classification considers pathological, clinical, and prognostic factors influencing treatment strategies.

#### 1.3.1. Myocardial Infarction Type 1

Type 1 MI results from atherosclerotic plaque rupture or erosion, leading to thrombus formation and myocardial necrosis. It is typically associated with coronary artery disease (CAD). Myocardial ischemia (clinical or ECG evidence) and elevated cardiac troponin (cTn) levels confirm the diagnosis. [10].

#### 1.3.2. Myocardial Infarction Type 2

Type 2 MI occurs due to an imbalance between oxygen supply and demand without plaque rupture [11]. Causes include coronary atherosclerosis, coronary spasm, microvascular dysfunction, embolism, or dissection. Non-cardiac factors like anemia, hypoxemia, or hypertension can also be involved [12].

#### 1.3.3. Myocardial Infarction Type 3

Type 3 MI is suspected when patients present signs of ischemia (e.g., ECG changes or ventricular fibrillation) but die before cardiac biomarkers can be assessed or found elevated. These patients are classified as having type 3 myocardial infarction when there is a high suspicion of an acute myocardial ischemic event, even in the absence of cardiac biomarker evidence of MI [12].

#### 1.3.4. Myocardial Infarction Type 4

Type 4 MI occurs during or after percutaneous coronary intervention (PCI) due to procedural complications, such as stent thrombosis or restenosis. Determining the extent to which any increase in cTn levels in a patient with acute MI is attributable to the MI itself versus the procedure can be challenging [13,14].

#### 1.3.5. Myocardial Infarction Type 5

Type 5 MI is associated with coronary artery bypass grafting (CABG) and reflects myocardial injury from ischemia or surgical trauma. Post-CABG cTn elevations are common, but ischemia is confirmed with ECG, angiographic, or imaging evidence. Routine ST and T wave changes after CABG are less reliable for diagnosing ischemia [15,16].

MI with non-obstructive coronary arteries (MINOCA) is caused by a heterogeneous group of vascular or myocardial disorders and can even be linked to substance abuse. This condition is critical because, unlike traditional myocardial infarction, it is not caused by obstructive plaques in the coronary arteries [17]. Additionally, MINOCA remains a critical subject of interest because it is associated with a higher risk of adverse outcomes, particularly reinfarction during follow-up, if underlying risk factors such as drug abuse are not addressed [18].

## 2. Drug-Induced Myocardial Infarction

**Figure 1:**
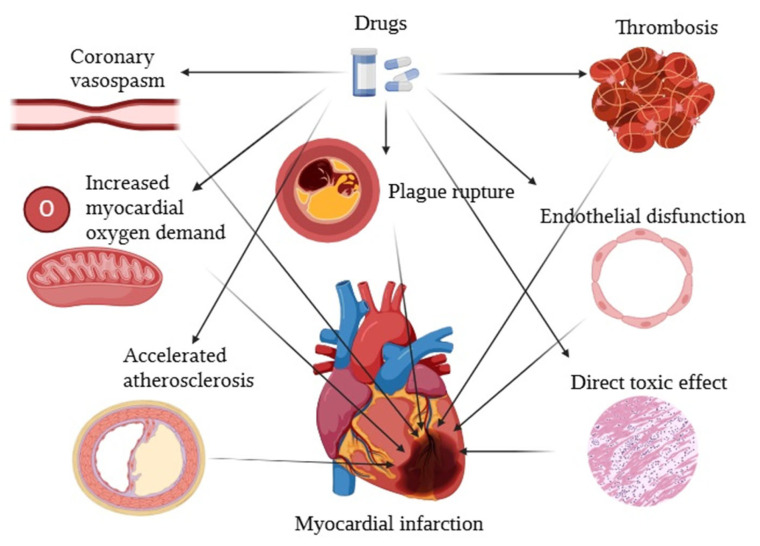
Pathophysiological mechanisms of drug-induced myocardial infarction.

Drug-induced MI refers to myocardial cell death caused by the use of certain substances, including both prescribed medications and illicit drugs. These substances can lead to MI through various mechanisms, including coronary vasospasm, increased myocardial oxygen demand, endothelial dysfunction, accelerated atherosclerosis, plaque rupture, thrombosis, or direct toxic effects on the myocardium, as shown in Figure 1. The pathophysiological mechanisms depend not only on the nature of the drug involved but also on the underlying cardiovascular condition of the patient [19].

The pathogenesis of coronary vasospasm is multifaceted and can be classified into three primary mechanisms: endothelial dysfunction in the intimal layer, hyperreactivity of vascular smooth muscle cells in the medial layer, and inflammation in the adventitia and perivascular adipose tissue [20].

Increased myocardial oxygen demand that involves an imbalance between oxygen supply and demand can also lead to MI when there is an increased myocardial workload in conditions such as sustained tachyarrhythmias, severe hypertension, or left ventricular hypertrophy that elevate the heart’s workload, necessitating higher oxygen consumption and an impaired oxygen supply if there is microvascular dysfunction, vasospasm, or inadequate coronary perfusion due to low diastolic blood flow [21].

Certain drugs may induce lipid metabolism disturbances, such as hyperlipidemia and altered low-density lipoprotein (LDL) oxidation, leading to foam cell formation and lipid plaque deposition. Direct vascular smooth muscle cell proliferation and migration can also contribute to plaque growth and instability. Chronic oxidative stress and systemic inflammation further amplify these effects, accelerating the progression of atherosclerosis and increasing the risk of cardiovascular events [22].

Coronary thrombosis occurs when certain medications or substances disrupt the delicate balance between pro-thrombotic and anti-thrombotic factors in coronary circulation. It can result from increased levels of coagulation factors (e.g., factor VII and fibrinogen) and reduced natural anticoagulants, such as protein C and antithrombin III [23,24], or the stimulation of platelet activation through serotonin release or adrenergic receptor activation [25,26].

Endothelial dysfunction involves the reduced bioavailability of vasodilators, especially nitric oxide (NO), and increased endothelium-derived contracting factors. This imbalance impairs endothelium-dependent vasodilation and promotes endothelial activation characterized by pro-inflammatory, proliferative, and pro-coagulatory tendencies, favoring atherogenesis [27].

Myocardial injury due to oxidative stress produces irreversible damage to the cardiomyocyte membrane and changes in contractile proteins and mitochondria, which are the pathophysiological mechanisms of direct cardiotoxic effects observed in drugs [28] (Table 1).

Plaque rupture typically happens at the thinnest part of the cap, most infiltrated by foam cells (macrophages). The thinning of the fibrous cap is likely due to two concurrent mechanisms: the gradual loss of smooth muscle cells from the cap and the degradation of the collagen-rich cap matrix by infiltrating macrophages [29].

### 2.1. Nonsteroidal Anti-Inflammatory Drugs (NSAIDs)

Nonsteroidal anti-inflammatory drugs (NSAIDs) are a heterogeneous category of chemical compounds with varied structures but similar analgesic, antipyretic, and anti-inflammatory effects [30]. They are one of the most commonly used classes of drugs for relieving mild to moderate pain, often being administered without a medical prescription [31]. They can be classified based on their mechanism of action, chemical structure, and selectivity for inhibiting cyclooxygenase (COX) enzymes. Classification based on COX selectivity is as follows: (a) non-selective COX inhibitors (ex. ibuprofen, naproxen, diclofenac, and indomethacin), which inhibit both COX-1 and COX-2 enzymes, affecting prostaglandin synthesis throughout the body and (b) COX-2 selective inhibitors or coxibs (celecoxib, etoricoxib, rofecoxib, rofecoxib), which selectively inhibit the COX-2 enzyme, reducing inflammation while theoretically having a lesser impact on the gastrointestinal tract [32].

The risk of myocardial infarction is increased with most NSAIDs, especially in patients with a history of heart disease or those with a high cardiovascular risk [33]. Cardiovascular adverse reactions of NSAIDs, even of MI, are favored, on the one hand, by the impairment of the physiological platelet aggregation process and, on the other hand, by increased blood pressure due to water and Na+ retention, exacerbating hypertension. This increase in blood pressure can elevate myocardial oxygen demand, potentially triggering cardiovascular ischemic events in susceptible individuals [33].

One of the factors associated with the increased risk of MI for both nonselective agents and coxibs is the degree of cyclooxygenase-2 (COX-2) inhibition. Selective COX-2 inhibition is associated with the reduced endothelial synthesis of prostaglandin I2 (PGI2), which has vasodilator and antiplatelet effects, without affecting the synthesis of thromboxane (TX), which has vasoconstrictive and pro-aggregate effects on platelets. Furthermore, the reduction in PGI2 activity could also predispose to endothelial damage [34]. In susceptible patients, selective COX-2 inhibition may result in an increased infarcted area, a significant thinning of the ventricular wall in the affected area, and an increased risk of myocardial wall rupture [35].

While most NSAIDs have been associated with an increased risk of MI, naproxen appears to have a comparatively lower risk than other agents. Nonetheless, the use of any NSAID should be carefully considered in patients with a history of cardiovascular disease or those at high cardiovascular risk [36].

### 2.2. Hormonal Therapies

Hormonal imbalances or hormone therapy can influence cardiovascular risk through a variety of mechanisms. Hormone-induced MI refers to situations when endogenous or exogenous hormones contribute to the development of myocardial ischemia and infarction.

Estrogen is a very important sexual hormone that has important and complex cardiovascular effects. In premenopausal women, it is generally considered to have cardioprotective effects as it promotes vasodilation through enhanced nitric oxide (NO) production and maintains favorable lipid profiles. However, postmenopausal estrogen decline is associated with increased cardiovascular risk as a result of the atherosclerotic process [37].

Estrogen can reduce atherosclerosis progression, but exogenous estrogen therapy has been linked to an increased risk of thromboembolic events and MI, especially in older women or those with pre-existing cardiovascular risk factors. For instance, oral estrogen administration may increase the risk of blood clot formation due to activated protein C resistance and the stimulation of the liver to produce matrix metallic protease 9. This alters atherosclerotic plaque formation and rupture, leading to MI type 1 [38].

Estrogens are a key component in various contraceptive methods, including combined oral contraceptive pills, combined injectable contraceptives, contraceptive vaginal rings, transdermal contraceptive patches, and combined emergency contraceptive pills. These formulations are termed “combined contraceptives” because they include both estrogen and a form of progestin [39].

Testosterone is another important sexual hormone that plays a role in cardiovascular health. In men, low testosterone levels are associated with adverse cardiovascular outcomes, whereas testosterone replacement therapy has raised concerns about potential cardiovascular risks [40]. Recently, it was observed that short-term testosterone therapy increased the risk of mortality and cardiovascular events in men over the age of 65 years, while longer-term therapy seemed to be associated with a reduced risk of adverse cardiovascular events, mortality, and also of prostate cancer [41].

Testosterone influences cardiovascular health through its effects on lipid metabolism, glucose homeostasis, and inflammation [42]. It may promote coronary artery vasodilation, but testosterone therapy has been variably associated with increased thrombotic risk, and it is controversial. Investigations of global coagulation in the setting of thrombosis need to be improved [43].

Thyroid hormones can influence cardiovascular health, and their imbalances, both hyperthyroidism and hypothyroidism, are associated with increased cardiovascular risk [44,45]. In hyperthyroidism, the elevated thyroid hormone levels induce tachycardia and increased cardiac output. In this clinical situation, the myocardial oxygen demand increases, putting more stress on the heart muscle [46]. Hyperthyroidism can cause systolic hypertension, increasing the workload on the heart and possibly leading to left ventricular hypertrophy, with secondary oxygen imbalance in the myocardial tissue [47]. Coronary artery vasospasm may also occur due to the hyperadrenergic state associated with hyperthyroidism, which can reduce blood flow to the myocardium, leading to ischemia and potentially type 2 MI [46].

Low thyroid hormone levels are associated with dyslipidemia, characterized by increased total cholesterol and low-density lipoprotein (LDL-col) levels. This can accelerate the process of atherosclerosis, leading to coronary artery stenosis [48]. Hypothyroidism can induce diastolic hypertension, which may promote the progression of coronary artery disease [49]. Additional endothelial dysfunction reported in hypothyroidism can impair vasodilation as a result of decreased NO production, contributing to reduced coronary blood flow and predisposing the individual to type 1 MI in case of plaque rupture [50].

Thyroid hormone imbalances, especially elevated thyroid hormone levels in hyperthyroidism, increase the risk of atrial fibrillation and other arrhythmias, which can lead to thromboembolism, including coronary artery embolism, resulting in MI [51].

### 2.3. Catecholamines

Catecholamines, which include adrenaline (epinephrine) and noradrenaline (norepinephrine), play a significant role in stress. Their clinical utility stems from their ability to modulate heart rate, blood pressure, and vascular tone, making them valuable drugs in treating cardiovascular emergencies and other acute conditions. However, their use must be carefully monitored due to potential adverse effects such as arrhythmias, increased myocardial oxygen demand, and potential ischemia [52].

Adrenaline binds to beta receptors and stimulates the sympathetic nervous system, increasing heart rate, blood pressure, and myocardial contractility. These changes elevate the myocardial oxygen demand, exacerbating any existing CAD and leading to an imbalance between oxygen supply and demand. The increased sympathetic activity induced by adrenaline can also lead to coronary vasospasm, triggering ischemia and myocardial infarction [53].

Adrenaline can significantly influence the thrombosis process and platelet function at clinically relevant concentrations in human blood. It can promote platelet activation, increasing phosphatidylserine (PS) exposure on their surface. PS exposure plays a crucial role in the coagulation process, facilitating the formation of coagulation factor complexes and thus enhancing thrombin generation and fibrin formation. In addition, the effects of adrenaline can change the structure of the fibrin clot, making it denser and more resistant to fibrinolysis. The inhibition of fibrinolysis and changes in clot structure may contribute to a prothrombotic status, increasing the risk of thrombus formation in the cardiovascular system [54]. These mechanisms are fundamental in individuals with predisposing factors for thrombosis or cardiovascular disease, where the impact of adrenaline on platelet activation and clot stability may exacerbate the risk of myocardial infarction or other thrombotic events. Commonly used antiplatelet drugs could be a reliable solution to reduce the prothrombotic state caused by the synergy between adrenaline and collagen [54].

High levels of circulating adrenaline may induce direct toxicity to cardiac muscle cells by causing calcium overload and oxidative stress. This cellular damage can manifest as myocardial injury or necrosis, even in the absence of significant coronary artery obstruction [55].

Noradrenaline stimulates the alpha- and beta-adrenergic receptors and is frequently used in intensive care settings, especially in managing septic shock, hypotension, and cardiac arrest. Its vasoconstrictive and heart-stimulating effects can help maintain adequate blood pressure and perfusion to vital organs [56].

Noradrenaline-induced MI can occur due to the effects of norepinephrine on the cardiovascular system, especially when administered in high doses or in individuals with predisposing conditions [57]. Noradrenaline, a potent vasoconstrictor, can cause the coronary arteries to constrict, decreasing blood flow to the myocardium and leading to an imbalance between oxygen supply and demand. This vasoconstriction is relevant in the presence of pre-existing CAD or endothelial dysfunction, which can lead to myocardial ischemia and eventually result in MI [58].

Like adrenaline, norepinephrine increases myocardial contractility even if it has minimal effects on the heart rate, resulting in higher oxygen consumption by the heart [59]. In cases where the coronary arteries cannot supply sufficient oxygen to meet this increased demand, myocardial ischemia may develop. Noradrenaline can significantly elevate blood pressure by causing systemic vasoconstriction. The increased afterload forces the heart to work harder to eject blood, which increases myocardial oxygen demand and can precipitate ischemic events, particularly in patients with underlying cardiac conditions [60].

High levels of norepinephrine can contribute to a pro-thrombotic state by increasing platelet aggregation. This increased risk of clot formation within the coronary arteries can promote acute coronary syndromes, including MI. The blockade of α-adrenergic receptors partially attenuated the effects of noradrenaline on coagulation and may have preventive potential in susceptible individuals [61]. Prolonged exposure to high levels of norepinephrine may cause direct damage to cardiac muscle cells by inducing apoptosis via protein kinase A activation and increased cytosolic Ca^2+^ influx, leading to necrosis. It can often be observed in critically ill patients receiving high doses of norepinephrine as a vasopressor [62].

### 2.4. Glucocorticoids

Glucocorticoids are a group of anti-inflammatory drugs that include physiological steroid hormones (secreted by the adrenal glands) and synthetic ones. They show marked anti-inflammatory properties and are often used in conditions like autoimmune diseases. Glucocorticoids also have important effects on carbohydrate and protein metabolism and, therefore, have been associated with increased cardiovascular risk [63].

The main natural corticoid is cortisol or hydrocortisone, which is produced in small amounts in the body and whose secretion is controlled by the hypothalamic-pituitary system via corticotrophin and corticotrophin-releasing hormone. Prednisone, prednisolone, methylprednisone, triamcinolone, and dexamethasone are synthetic corticosteroids [64].

Glucocorticoids favor sodium and water retention, leading to hypervolemia and, consequently, elevated blood pressure. Hypertension is a well-established risk factor for MI, as it can not only contribute to the development of atherosclerosis and increase the workload on the heart, but also can lead to plaque rupture when blood pressure is very high [65].

These drugs can cause dyslipidemia, characterized by elevated LDL cholesterol and triglycerides and decreased high-density lipoprotein cholesterol (HDL-col) [66]. These lipid changes contribute to atherosclerotic plaque formation and increase the risk of coronary artery occlusion [67].

Glucocorticoids can impair endothelial cell function by reducing the bioavailability of NO. This fact leads to increased vascular resistance and decreased elasticity, contributing to coronary artery disease. This endothelial dysfunction is linked to the pro-atherogenic and pro-thrombotic environment, enhancing the risk of myocardial infarction [68]. Even more, they can enhance platelet aggregation and increase the levels of clotting factors, which increases the risk of thrombus formation in the coronary arteries. This pro-thrombotic effect is partially due to the fact they can enhance platelet activity and the expression of coagulation proteins like fibrinogen [68]. These changes contribute to an increased risk of vessel occlusion and myocardial ischemia, particularly in patients with pre-existing cardiovascular risk factors or conditions that predispose to clot formation [68].

Elevating blood pressure increases the heart’s afterload, raising myocardial oxygen demand. Additionally, glucocorticoids can cause hyperglycemia, which not only further augments myocardial oxygen consumption but also contributes to endothelial dysfunction and accelerated atherosclerosis [69].

Overall, the risk of glucocorticoid-induced MI may be higher in individuals with pre-existing CAD or those receiving high-dose or long-term glucocorticoid therapy. Monitoring and managing cardiovascular risk factors in patients undergoing glucocorticoid treatment is important to minimize the likelihood of adverse cardiac events such as MI [70].

### 2.5. Anticoagulants

Anticoagulants are generally used to reduce the risk of thrombotic events, but they can paradoxically contribute to MI in some specific circumstances. These drugs affect hemostasis and reduce blood clotting, which can, in rare cases, exacerbate bleeding within an atherosclerotic plaque. Plaque hemorrhage can lead to its expansion, increased instability, and subsequent rupture, which may result in thrombus formation and the occlusion of the coronary artery [71].

Improper dosing can pose risks as a reduced dose of anticoagulation may not adequately prevent thrombosis, whereas excessive anticoagulation may cause bleeding complications that could lead to secondary cardiovascular events [71].

After discontinuing certain anticoagulants (such as warfarin or vitamin K antagonists), a rebound hypercoagulable state can occur. This rebound effect can increase the risk of thrombosis and potentially provoke an MI [72].

Heparin-induced thrombocytopenia (HIT) is a complication associated with heparin therapy. In this condition, the immune system forms antibodies against the complex of heparin and platelet factor 4, leading to platelet activation and increased thrombotic risk. This condition can cause arterial thrombosis and increase the risk of myocardial infarction [73].

Anticoagulant therapy requires careful monitoring to avoid these complications, particularly in patients with existing cardiovascular risk factors.

### 2.6. Insulin and Diabetic Hormonal Imbalance

Elevated insulin levels, characteristic of hyperinsulinemia, can contribute to endothelial dysfunction. Hyperinsulinemia can increase the production of pro-inflammatory cytokines, promote smooth muscle cell proliferation, and encourage the deposition of LDL-chol in arterial media, thereby accelerating atherosclerosis and increasing the risk of CAD and MI [74].

In type 2 diabetes, insulin resistance often accompanies hyperinsulinemia. This resistance can exacerbate dyslipidemia, hypertension, and other metabolic abnormalities, all of which are major risk factors for CAD and MI [75]. The chronic inflammatory state associated with insulin resistance also contributes to plaque instability, which can precipitate an MI [75].

While insulin therapy is essential for controlling blood glucose levels in diabetes, it can lead to episodes of hypoglycemia, especially if the dosage is not carefully monitored or the patients are not well instructed. Hypoglycemia has been associated with adverse cardiovascular outcomes, including arrhythmias, increased sympathetic nervous activity, and heightened platelet aggregation, which may increase the risk of acute cardiovascular events [76].

In addition to insulin, other hormones like glucagon, cortisol, and catecholamines can be dysregulated in diabetes, further influencing cardiovascular risk. Elevated cortisol levels can increase blood pressure and promote atherosclerosis, while high levels of catecholamines can exacerbate myocardial oxygen demand and contribute to ischemia [77].

Addressing these factors in diabetic patients involves glycemic control and managing other cardiovascular risk factors through lifestyle changes and pharmacotherapy.

### 2.7. Antipsychotic Medications

Second-generation antipsychotics (SGAs), such as olanzapine and risperidone, have been associated with an increased risk of cardiovascular events, including MI. The underlying mechanisms are primarily linked to their metabolic side effects [78].

SGAs are known to cause significant weight gain, which is an important cardiovascular risk factor. Excess weight, especially central obesity, increases the risk of developing conditions like hypertension, dyslipidemia, and insulin resistance, all of which are contributors to coronary artery disease and eventually to MI [79].

The long-term use of SGAs can negatively affect lipid metabolism, leading to elevated triglycerides and LDL cholesterol while reducing HDL cholesterol. These changes contribute to the development of atherosclerosis, which can increase the likelihood of MI [80].

Some SGAs can induce or worsen insulin resistance and cause hyperglycemia, even leading to new-onset diabetes [81], and they might elevate cardiovascular risk. They could be associated with MI, as described above.

These drugs may promote a pro-inflammatory state, contributing to endothelial dysfunction, plaque instability, and thrombosis, increasing the risk of an acute cardiovascular event like MI [80].

Addressing these risks involves the regular monitoring of weight, blood pressure, blood glucose, and lipid levels in patients taking SGAs. Lifestyle interventions and medications to manage these metabolic side effects may also help reduce the associated cardiovascular risks.

### 2.8. Cancer Treatments

Certain chemotherapy drugs, such as anthracyclines (e.g., Doxorubicin) and some targeted therapies (e.g., trastuzumab), are known to have cardiotoxic effects, which can elevate the risk of MI through various mechanisms [82].

Anthracyclines (e.g., Doxorubicin) are widely used in cancer treatment but can cause dose-dependent cardiotoxicity. Doxorubicin, in particular, generates reactive oxygen species (ROS) and induces oxidative stress, leading to direct myocardial damage. This process can disrupt mitochondrial function in cardiac cells, contribute to cardiomyocyte apoptosis, and promote fibrosis, potentially increasing the risk of heart failure and MI [83].

Trastuzumab, a monoclonal antibody used in treating HER2-positive breast cancer, can interfere with the HER2 signaling pathway in cardiomyocytes, which is crucial for cell survival and repair mechanisms. The disruption of this pathway may lead to decreased cardiac contractility and increased susceptibility to myocardial injury [84]. Although less common than anthracyclines, trastuzumab-related cardiotoxicity can manifest as reduced left ventricular ejection fraction and even heart failure, potentially increasing the risk of MI, mainly when used in combination with other cardiotoxic agents [85].

Chemotherapy drugs can promote a pro-inflammatory status and cause endothelial dysfunction, which may accelerate atherosclerosis and destabilize the existing coronary plaques. These factors increase the likelihood of thrombus formation, vessel occlusion, and subsequent MI [86].

Cardioprotective strategies such as monitoring cardiac function, dose limitations, the use of liposomal formulations of anthracyclines, and the administration of cardioprotective agents may be employed to mitigate these risks. Regular cardiac monitoring and the early detection of changes in heart function are essential for patients undergoing treatment with these chemotherapy drugs [87,88].

### 2.9. Other Medications

Calcineurin inhibitors, such as cyclosporine and tacrolimus, are immunosuppressive drugs commonly used to prevent organ transplant rejection. However, these medications can increase the risk of myocardial infarction MI through several mechanisms, such as hypertension, endothelial dysfunction, dyslipidemia, and prothrombotic state [89]. Calcineurin inhibitors often cause dose-dependent increases in blood pressure. The mechanism behind this hypertensive effect involves increased sympathetic nervous system activity, sodium retention, and reduced production of vasodilators like NO. Chronic hypertension resulting from calcineurin inhibitors can contribute to the development of atherosclerosis and eventually lead to MI [90]. Reduction in NO production can impair endothelial cell function, leading to increased vascular resistance, inflammation, and acceleration of the atherosclerotic process [91]. These drugs are also associated with lipid metabolism disturbances, including increased cholesterol and triglyceride levels [22]. Elevated lipid levels contribute to the formation and progression of atherosclerotic plaques, which can increase the likelihood of plaque rupture and subsequent MI [92]. Calcineurin inhibitors may increase the risk of thrombus formation by enhancing platelet reactivity and coagulation pathways. This can lead to coronary artery occlusion, especially in patients who already have underlying atherosclerotic plaques [93].

Due to these risks, close blood pressure, lipid levels, and cardiac function monitoring are recommended in patients receiving calcineurin inhibitors. Adjusting the dose, using antihypertensive therapies, or switching to less cardiotoxic immunosuppressants may help mitigate the cardiovascular risks associated with these drugs [94].

Erythropoiesis-stimulating agents (ESAs), such as erythropoietin, are used to treat anemia, especially in patients with chronic kidney disease (CKD) or those undergoing chemotherapy. While effective in raising hemoglobin levels and reducing the need for blood transfusions, ESAs have been associated with an increased risk of cardiovascular events [95]. ESAs stimulate red blood cell production, which can elevate blood viscosity. Higher blood viscosity may impair microcirculatory flow and increase the risk of thrombus formation, potentially leading to coronary artery occlusion and MI [96]. These drugs can elevate blood pressure as a side effect, possibly due to increased blood volume and changes in vascular tone, as they might promote endothelial dysfunction [97].

It was reported that aiming for higher hemoglobin targets with ESA therapy can be linked to increased cardiovascular risks. Patients treated to achieve near-normal hemoglobin levels experienced more adverse cardiovascular events compared to those maintained at lower target levels [98]. The cardiovascular risks associated with ESAs highlight the need for careful dosing, individualized treatment goals, and monitoring of hemoglobin levels, especially in patients with existing CAD [98].

Triptans are commonly used to treat migraine headaches. They work by stimulating serotonin (5-HT) receptors, specifically the 5-HT1B and 5-HT1D receptors, leading to the constriction of blood vessels in the brain to relieve migraine symptoms. However, their vasoconstrictive properties can also affect coronary arteries, potentially causing coronary artery vasospasm in susceptible individuals [98]. This vasospasm can reduce blood flow to the heart muscle, potentially leading to myocardial ischemia or even MI, especially in individuals with existing cardiovascular risk factors or undiagnosed coronary artery disease [99].

Several case reports and studies have documented the occurrence of triptan-induced coronary artery vasospasm, which can manifest as chest pain, shortness of breath, or more severe ischemic events [99,100,101,102,103,104]. Because of these potential risks, triptans are contraindicated in individuals with a history of coronary artery disease, peripheral vascular disease, uncontrolled hypertension, or significant cardiovascular risk factors [105].

Isoproterenol, a synthetic catecholamine, and β-adrenergic agonist can induce myocardial infarction experimentally by excessively stimulating β1 and β2 receptors [106]. This stimulation increases heart rate, contractility, and myocardial oxygen demand [107]. At high doses, isoproterenol causes intense myocardial strain and an imbalance between oxygen supply and demand, resulting in myocardial ischemia and necrosis [108]. Furthermore, it promotes oxidative stress, lipid peroxidation, and calcium overload in cardiac cells, exacerbating myocardial injury [109]. For these reasons, isoproterenol is often used in animal models to study myocardial infarction mechanisms and potential treatments [106,107,108,109,110,111,112].

Among antibiotics, macrolides, particularly erythromycin and clarithromycin, have been linked to an increased risk of myocardial infarction. The mechanism of the association between macrolides and MI remains unclear. Clarithromycin has been reported to activate macrophages, potentially destabilizing plaques through an inflammatory cascade, thereby increasing the risk of MI. Unlike azithromycin, clarithromycin inhibits cytochrome P450 enzymes, significantly raising the potential for drug–drug interactions. These interactions can impair the biotransformation and antiplatelet activity of medications like clopidogrel, which are critical in treating and preventing coronary heart disease [113].

### 2.10. Stimulants and Recreational Drugs

Cocaine is a well-documented trigger for MI, primarily due to its potent vasoconstrictive effects on coronary arteries. This intense vasoconstriction (due to norepinephrine and dopamine reuptake inhibition) can lead to coronary artery spasms, which reduce or completely obstruct blood flow to the heart muscle, precipitating an MI [114]. Additionally, cocaine increases heart rate and blood pressure, which elevates myocardial oxygen demand. When combined with the restricted blood flow from coronary artery spasms, this imbalance significantly heightens the risk of myocardial infarction [115].

Amphetamines and methamphetamines are stimulants that can markedly elevate heart rate and blood pressure, increasing the workload on the heart. This elevated cardiovascular stress raises myocardial oxygen demand, which, combined with stimulant-induced vasoconstriction, can lead to myocardial ischemia [116]. Over time, chronic use can lead to structural heart changes, including left ventricular hypertrophy and an increased risk of coronary artery disease [116]. In acute cases, the surge in catecholamines can precipitate MI, even in younger individuals without pre-existing coronary artery disease [117].

Ecstasy (MDMA) can indeed pose serious cardiovascular risks, mainly due to its stimulant effects, which increase heart rate and blood pressure significantly [118]. These hemodynamic changes increase the heart’s oxygen demand and stress, which, coupled with the potential for coronary artery spasm, can lead to ischemia and even MI [118]. MDMA can also trigger hyperthermia and dehydration, further straining the cardiovascular system and potentially worsening the risk of adverse cardiac events [118].

Nicotine is a potent vasoconstrictor that raises blood pressure and heart rate, increasing myocardial oxygen demand. Additionally, chronic nicotine exposure from smoking accelerates atherosclerosis by promoting endothelial dysfunction and inflammation within coronary arteries [118]. The atherosclerotic plaques narrow the coronary arteries, raising the risk of plaque rupture and thrombosis, which can trigger MI [119]. The combined effects of vasoconstriction and atherosclerosis make nicotine a significant risk factor for MI, especially in long-term smokers [120].

Opioids, such as morphine, heroin, and synthetic opioids, can contribute to MI through several indirect mechanisms. They often cause vasodilation and hypotension, which can lead to reduced coronary perfusion. In susceptible individuals, this can precipitate myocardial ischemia, particularly in the setting of pre-existing coronary atherosclerosis [121]. A hallmark of opioid overdose is respiratory depression, which leads to hypoxemia [122]. Decreased oxygen levels can impair myocardial oxygen supply, creating an imbalance between oxygen supply and demand and potentially causing ischemic injury. The delayed gastrointestinal absorption of P2Y12 inhibitors due to opioids and the consequent impairment in antiplatelet activity can also lead to MI [123].

Cannabis use, particularly at high doses or in vulnerable populations, has been associated with MI. Although the exact pathophysiological mechanisms remain under investigation, several plausible pathways link cannabis to acute coronary events. Cannabis can cause a transient increase in heart rate and systemic blood pressure, particularly immediately after use, and these hemodynamic changes elevate myocardial oxygen demand [124]. Cannabis is known to induce coronary vasospasm, likely through the activation of the endocannabinoid system or sympathetic stimulation. Vasospasm can reduce coronary blood flow, leading to ischemia or infarction, even in individuals without significant atherosclerotic disease [125]. Regular cannabis use has been linked to endothelial dysfunction, which impairs vasodilation and promotes a pro-inflammatory state. This dysfunction may increase the risk of atherosclerosis and plaque destabilization, contributing to MI [125]. Cannabis may have pro-thrombotic properties, increasing the risk of thrombus formation in coronary arteries. This effect could be mediated by alterations in platelet function or coagulation pathways [126].

### 2.11. Kounis Syndrome

Kounis syndrome is characterized by the simultaneous occurrence of acute coronary syndromes—such as coronary spasm, acute myocardial infarction, and stent thrombosis—in the context of allergic or hypersensitivity reactions, including anaphylactic or anaphylactoid events. It involves mast cell and platelet activation, along with the participation of interrelated inflammatory cells such as macrophages and T-lymphocytes. The syndrome is driven by the release of inflammatory mediators, including histamine, platelet-activating factor, arachidonic acid derivatives, neutral proteases, and various cytokines and chemokines during the allergic activation process [127,128]. 

Kounis syndrome can be triggered by various drugs, foods, environmental factors, and clinical conditions. Commonly implicated drugs include widely used medications such as aspirin, antihypertensives, corticosteroids, antibiotics, and nonsteroidal anti-inflammatory drugs, which constitute some of the main offenders [129]. 

The diagnosis of this syndrome relies on clinical symptoms and signs, along with laboratory, electrocardiographic, echocardiographic, and angiographic evidence. These findings, often accompanied by allergic symptoms, help in making the correct diagnosis. Recently, advanced diagnostic tools such as cardiac magnetic resonance imaging and myocardial scintigraphy have proven valuable in confirming the condition. Consequently, patients presenting with systemic allergic reactions alongside the clinical, electrocardiographic, and laboratory indicators of acute myocardial ischemia should be evaluated for the possibility of Kounis syndrome [130].

The treatment of Kounis syndrome primarily involves managing the allergic or anaphylactic reaction, followed by stabilizing the coronary vasculature using established medical or interventional approaches. Initial therapies typically include corticosteroids, epinephrine, and antihistamines, administered until the desired therapeutic effect is achieved [131].

### 2.12. Management of Drug-Induced Myocardial Infarction

Management of drug-induced MI requires the rapid identification of the causative drug, mitigation of its effects, and tailored treatment to address both the acute event and its underlying pathophysiology.

The immediate cessation of the drug suspected of causing the MI and substituting with an alternative medication, if clinically necessary, is crucial to prevent further myocardial injury or aggravation [126].

Following acute coronary syndrome management protocols, the first steps in acute MI include oxygen therapy if hypoxemia is present, nitrates to relieve ischemic chest pain (unless contraindicated), dual antiplatelet therapy (DAPT), anticoagulation (e.g., heparin) to reduce thrombus formation, beta-blockers (in stable patients without contraindications) to reduce oxygen consumption, and high-dose statins to stabilize atherosclerotic plaques [132].

In the case of cocaine- or amphetamine-induced MI, the administration of beta-blockers should be avoided due to the risk of unopposed alpha-adrenergic activity, which can worsen vasoconstriction [133]. The administration of benzodiazepines can be helpful to reduce adrenergic stimulation and anxiety [134,135].

Advanced interventions, such as percutaneous coronary intervention (PCI) or thrombolysis, should be considered for patients with persistent ischemia depending on availability and timing. In severe or refractory cases, a coronary artery bypass graft (CABG) may be [132].

Regarding the specific mechanisms of drug-induced MI, vasospasm can be treated with calcium channel blockers (e.g., nifedipine and diltiazem) [136]. At the same time, nitrates can be helpful in relieving vasospastic episodes caused by drugs like cocaine, amphetamines, or cannabis [137]. Initial DAPT with aspirin and a P2Y12 inhibitor (e.g., clopidogrel and ticagrelor) is recommended to reduce platelet aggregation and thrombosis. Glycoprotein IIb/IIIa inhibitors should be considered for high thrombus burden [132].

The long-term management of patients after MI includes cardiovascular risk reduction and patient education. First, informing the patient about the risks of the causative drug and providing alternatives or modification dosages of essential medications to reduce future risks should be considered. Lifestyle changes such as smoking cessation, dietary modifications, and regular physical activity are also recommended. Chronic treatment with statins is recommended for lipid management. Angiotensin-converting enzyme inhibitors (ACE inhibitors) and angiotensin II receptor blockers (ARBs) are helpful for hypertension treatment and for the prevention of left ventricular remodeling after MI [138,139]. Beta-blockers are recommended for chronic ischemic control and the secondary prevention of myocardial infarction and heart failure [140].

Psychosocial support offered by addiction specialists is mandatory in care for patients with abuse of stimulants and recreational drugs [141].

## 3. Conclusions

In conclusion, drug-induced myocardial infarction represents a significant clinical concern, stemming from various pharmacological agents that can provoke or exacerbate myocardial ischemia and infarction through multiple mechanisms. These include coronary vasospasm, endothelial dysfunction, accelerated atherosclerosis, imbalance between oxygen supply and demand, or direct cardiotoxic effects. Both therapeutic substances, such as NSAIDs, hormonal treatments, catecholamines, glucocorticoids, chemotherapy drugs, and antipsychotic medications, and illicit drugs like cocaine, amphetamines, or methamphetamines and ecstasy, have been implicated in increasing the risk of MI. Pathophysiological mechanisms often depend on the specific properties of the drugs and the individual’s underlying cardiovascular health.

Recognizing drug-induced MI as a distinct entity is crucial for prompt diagnosis and tailored management. Preventive strategies should focus on identifying high-risk patients, optimizing the use of medications with known cardiotoxic potential, and carefully monitoring the cardiovascular system during therapy. Understanding the underlying pathophysiological mechanisms not only aids in managing acute episodes but also helps guide long-term prevention efforts aimed at reducing cardiovascular risk. Future research should continue to explore the complex interactions between pharmacological agents and myocardial health, aiming to minimize the occurrence of adverse cardiac events associated with drug therapy.

## Figures and Tables

**Table 1:** Main pathophysiological mechanisms of drug-induced myocardial infarction and involved drugs.

Coronary Vasospasm	Increased Myocardial Oxygen Demand	Accelerated Atherosclerosis	Thrombosis	Endothelial Disfunction	Direct Toxic Effect	Plaque Rupture
Medications						
Thyroid hormones (high levels) Adrenaline Noradrenaline Triptans	Nonsteroidal anti-inflammatory drugs *(NSAIDs)* Thyroid hormones (high levels) Adrenaline Noradrenaline Glucocorticoids Isoproterenol	Glucocorticoids Insulin Second-generation antipsychotics Chemotherapy drugs Calcineurin inhibitors	Nonsteroidal anti-inflammatory drugs *(NSAIDs)* Estrogen Testosterone Thyroid hormones (high levels) Adrenaline Noradrenaline Glucocorticoids Anticoagulants Second-generation antipsychotics Chemotherapy drugs Erythropoiesis-stimulating agents Antibiotics	Nonsteroidal anti-inflammatory drugs *(NSAIDs)* Thyroid hormones (low levels) Glucocorticoids Insulin Second-generation antipsychotics Chemotherapy drugs Calcineurin inhibitors Erythropoiesis-stimulating agents	Adrenaline (high doses) Noradrenaline Chemotherapy drugs Isoproterenol	Estrogen Glucocorticoids Anticoagulants Calcineurin inhibitors Antibiotics
Stimulants and Recreational Drugs						
Cocaine Amphetamines and methamphetamines Ecstasy Nicotine Cannabis	Cocaine Amphetamines and methamphetamines Ecstasy Nicotine Opioids Cannabis	Nicotine	Opioids Cannabis	Nicotine Cannabis		Cannabis

## Data Availability

No new data were created or analyzed in this study.
